# Spatial Regulation of Membrane Fusion Controlled by Modification of Phosphoinositides

**DOI:** 10.1371/journal.pone.0012208

**Published:** 2010-08-17

**Authors:** Fabrice Dumas, Richard D. Byrne, Ben Vincent, Tina M. C. Hobday, Dominic L. Poccia, Banafshé Larijani

**Affiliations:** 1 Cell Biophysics Laboratory, Lincoln's Inn Fields Laboratories, Cancer Research UK, London, United Kingdom; 2 CNRS, IPBS (Institut de Pharmacologie et de Biologie Structurale), Toulouse, France; 3 Université de Toulouse, UPS, IPBS (Institut de Pharmacologie et de Biologie Structurale), Toulouse, France; 4 Department of Biology, Amherst College, Amherst, Massachusetts, United States of America; University of California, Berkeley, United States of America

## Abstract

Membrane fusion plays a central role in many cell processes from vesicular transport to nuclear envelope reconstitution at mitosis but the mechanisms that underlie fusion of natural membranes are not well understood. Studies with synthetic membranes and theoretical considerations indicate that accumulation of lipids characterised by negative curvature such as diacylglycerol (DAG) facilitate fusion. However, the specific role of lipids in membrane fusion of natural membranes is not well established. Nuclear envelope (NE) assembly was used as a model for membrane fusion. A natural membrane population highly enriched in the enzyme and substrate needed to produce DAG has been isolated and is required for fusions leading to nuclear envelope formation, although it contributes only a small amount of the membrane eventually incorporated into the NE. It was postulated to initiate and regulate membrane fusion. Here we use a multidisciplinary approach including subcellular membrane purification, fluorescence spectroscopy and Förster resonance energy transfer (FRET)/two-photon fluorescence lifetime imaging microscopy (FLIM) to demonstrate that initiation of vesicle fusion arises from two unique sites where these vesicles bind to chromatin. Fusion is subsequently propagated to the endoplasmic reticulum-derived membranes that make up the bulk of the NE to ultimately enclose the chromatin. We show how initiation of multiple vesicle fusions can be controlled by localised production of DAG and propagated bidirectionally. Phospholipase C (PLCγ), GTP hydrolysis and (phosphatidylinsositol-(4,5)-bisphosphate (PtdIns(4,5)P_2_) are required for the latter process. We discuss the general implications of membrane fusion regulation and spatial control utilising such a mechanism.

## Introduction

Membrane fusion is required for many cell processes from vesicular transport to nuclear envelope reconstitution at mitosis. Historically, the role of lipids and lipid modifications in fusion has been based on model membranes in which an intermediate or hemifusion state, promoted by the localised reorganisation of lipids of negative curvature, leads to a transient fusion pore and eventually to complete fusion [1]. Recent work has integrated roles for both protein signalling and lipid modification in natural membrane fusion [1], [2], [3], [4], [5].

We have isolated a natural membrane vesicle fraction (MV1) from cytoplasm of fertilised oocytes. This membrane population consists of >50% phosphoinositides, is >100-fold enriched in a phosphatidylinositol-specific phospholipase C (PI-PLCγ) and is essential for membrane fusion leading to nuclear envelope formation [3], [6]. Using cell-free oocyte extracts to assemble nuclear envelopes from fusion of discrete membrane vesicle populations [7], we have shown that the early signalling events involve activation of a tyrosine kinase [8] which in turn activates PLCγ in MV1 [3]. Subsequent formation of diacylglycerol (DAG) alters the lamellar structure of these precursor membranes, facilitating their fusion with the endoplasmic reticulum (ER)-derived membranes that contribute most of the nuclear envelope [3], [5], [9].

Here we show, using FRET by two-photon FLIM and three dimensional reconstructions of immobilised nuclei, that the two pole regions to which MV1 membrane vesicles bind are the sites of initiation of fusion with adjacent (ER) membranes, and that further fusion propagates away from the poles to complete enclosure of the chromatin. Using inhibitors, we show that this process is dependent on PtdIns(4,5)P_2_, PLC and GTPase activity. We discuss how spatial control of membrane fusion may be regulated by regional binding of a potentially fusogenic membrane vesicle population and the novel consequences of such a mechanism.

## Materials and Methods

### Buffer and reagents

*Lytechinus pictus* sea urchins were purchased from Marinus (Long Beach, CA), 4-heptadecyl-7-hydroxycoumarin, BODIPY-C12 and DiIC12, from Invitrogen, U73122 and U73343 from Calbiochem, GTPγ[S] (guanosine 5-[γ-thio]triphosphate) from Sigma, and caged-GTP from Jena Bioscience. Recombinant SKIP proteins (Skeletal muscle and Kidney enriched Inositol Phosphatase, a phosphoinositide 5-phosphatase) were a generous gift from E. Rosivatz and R. Woscholski. Nuclear preparation buffer (SXN), TN (Tris/NaCl buffer) and egg lysis buffer [10] were prepared as described previously [11]. DABCO antifade was from Sigma and prepared at 2.5% (w/v) in LB. The ATP-generating system (ATP-GS) is 1 mM ATP, 20 mM creatine phosphate and 1 mg/ml creatine kinase in LB.

### Nuclei and egg extracts

Isolation and permeabilisation of sperm nuclei were adapted from methods described previously [7], [12]. Nuclei demembranated with 0.1% Triton X-100 were resuspended in freezing buffer [SXN supplemented with 0.16% (w/v) BSA and 16.5% (v/v) glycerol], frozen in liquid nitrogen and stored at −80°C. S10 cytoplasmic (G1 phase) extracts from eggs at 10 min post fertilization, membrane vesicles (MVs) and subfractions MV1 and MV2, and 150,000 g supernantant cytosolic egg extracts (S150) were prepared as previously described [13]

### Fluorescent labelling

Demembranated nuclei were incubated 1 hour at 4°C with 5 mM hydroxycoumarin in TN buffer to label nuclear envelope remnants (NERs). Nuclei were then collected by centrifugation (1000 g, 2 min.). Stock solutions of fluorescent probes were prepared in Wesson Oil (BODIPY-C12, 20 mM) or in MeOH (DiIC12, 10 mM). The amount of lipid was measured by phosphorus titration before adding the fluorophores in order to ensure that the probe/lipid ratio in the resulting vesicles was less than 1 mole %. MV0, MV1 or MV2 vesicles were mixed with fluorescent probes and vortexed for 5 min at room temperature. The samples were then centrifuged at 100,000 g for 30 min to remove the non-inserted fluorescent probes and resuspended in S150 cytosol.

### Binding and fusion assays

To a 1.5 ml Eppendorf tube, 10 µl of BODIPY-C12 labelled MVs, 10 µl of diIC12 labelled MVs in S150, 1.2 µl of ATP-generating system and 2 µl of demembranated sperm nuclei were added. The mixture was incubated at room temperature for 1 h. The unbound vesicles were removed by centrifugation through 0.5 M sucrose (1000 g, 3 min.) and the purified nuclei with bound vesicles were suspended in 4 µl S150 cytosolic egg extract supplemented with 1 mM caged-GTP, 1 µl of 2.5% DABCO and 5 µl 1% low melting point agarose at 30°C. The mixture was immediately mounted on a Mattek® dish and viewed under a 100X oil-immersion objective. Binding was confirmed by surface coating of fluorescent membranes on nuclei. Fusion was triggered by UV (Hg lamp) illumination of the sample for 2 seconds inducing the photoactivation of the GTP. Lifetime of the BODIPY-C12 (donor) was measured before (t = 0) and after GTP activation (t = 5, 15, 30, 45 and 60 minutes). Hydroxycoumarin fluorescence of NERs was recorded during activation by the Hg source. U73122 (30 µM), U73343 (30 µM) and GTPγ[S] (2 mM) inhibitors were applied just before mounting the samples on Mattek® dishes and fusion was initiated 15 min after according to Byrne et al (2005) [14]. Caged GTP was used in all experiments except GTPγ[S] assays. Alternatively, MVs were incubated with SKIP purified protein for 1 hour at room temperature prior to binding. The activity of the purified proteins was checked according to Schmid et al. (2004) [15]. Each experiment was repeated a minimum of three times (n = 3). The images are a representative of one experiment.

### Fluorescence lifetime imaging microscopy (FLIM)

All FLIM measurements were undertaken with a modified TE 2000-E inverted microscope. Fluorescence lifetime measurements were performed with an SPC 830 time-correlated single photon counting (TCSPC) electronic card (Becker and Hickl, Germany). A mode-locked tuneable Ti-sapphire laser (Mira 900; Coherent) pumped by a solid-state diode laser (Verdi; Coherent) was used. For two-photon excitation of BODIPY-C12, the laser was tuned at 890 nm and pumped at 6W. The Ti-sapphire laser generates 125-fs pulses with a repetition rate of 76.26 MHz and an average power output of 450 mW. The laser beam was focused with a 100X oil immersion objective lens (Nikon). Fluorescence was detected through the same objective in a descanned configuration with a fast photomultiplier (Hamamatsu 7400) after filtering with a bandpass filter (510–610 nm, Chroma Technology Corp). Acquisition times of the order of 60 s at low excitation power were used to achieve sufficient photon statistics for fitting (*i.e.* 100–10000 photons per pixel), while avoiding either pulse pile-up or photobleaching. Epifluorescence intensity images of both donor and acceptor were acquired with the mercury lamp source of the TE 2000-E microscope and fluorescence detected by a cooled CCD camera (Hamamatsu ORCA-ER). The cubes set in the TE 2000-E microscope turret were FITC (Nikon Ltd.) for BODIPY-C12 and G-2A (Nikon Ltd.) for the diIC12.

## Results

The cell-free assay to assemble nuclear membranes consists of a cytoplasmic extract of fertilised eggs (S10) and sperm nuclei demembranated with 0.1% Triton X-100, which leaves remnants of the sperm nuclear envelope at the tip and base of the nucleus. MV1 binds exclusively to these regions [7], [16], Supporting Information (Fig S1). The majority of bound vesicles however are derived from the ER (MV2), which bind over the entire surface, not just at the poles [17].

**Figure 1:**
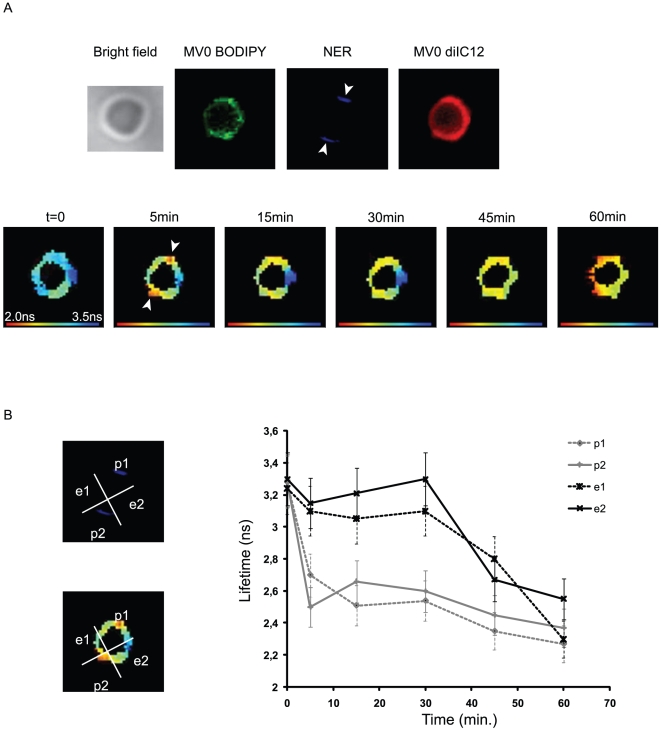
GTP-induced fusion is bi-polarised. (A) S10s containing total MVs (MV0) were independently labelled with either BODIPY-C12 (donor) or diIC12 (acceptor) and mixed together. Sperm nuclei and ATP-GS (ATP) were added. Nuclear envelope remnants of the nuclei were pre-labelled with hydroxycoumarin. Epifluorescence patterns of labelled nuclei with bound MVs were visualised by phase contrast and two-photon fluorescence microscopy using a 100X objective. MVs were bound around the entire periphery of the nucleus. The nuclear envelope remnants mark the former apex and base of the sperm nucleus (white arrowheads). Fluorescence lifetime of BODIPY was measured before (t = 0) and after (t = 5, 15, 30, 45 and 60 minutes) the induction of NE formation by photo activation of caged-GTP. (B) Quantification of FRET FLIM images. For analyses, nuclei were divided in four quadrants: p1 and p2 correspond to the poles of the nuclei that include NER while e1 and e2 correspond to the equatorial regions. The averaged mean lifetime was for each quadrant was plotted for each time point showing that MVs fusion is initiated in the polar quadrants and propagates toward the equator. Errors bars correspond to the standard deviation from 7 independent experiments.

Taking advantage of this difference, we initially labelled two sets of total membrane vesicles from S10 (MV0s), one with a donor fluorophore (BODIPY-C12) and the other with an acceptor fluorophore (DiIC12). The characterisation of these is described in MethodsS1. Nuclear envelope remnants (NER) were labelled with hydroxycoumarin (arrows, Fig. 1A). Since FRET is only possible between molecules that are in close proximity (1–10 nm), it is a reliable indicator of membrane fusion which permits the donor and acceptor to interact within a common continuous bilayer.

The nuclei were mounted on a cover slip with low-melting agarose to prevent movement prior to initiation of membrane fusion and confocal imaging. To accurately set the time of initiation, 2 mM caged GTP was included prior to embedding in agarose and after photo-activation by a UV source, fusion kinetics were assessed by the decrease in lifetime of the donor.

A reference point at t = 0 was taken in the absence of GTP (Supporting Information Movie S1). By 5 minutes post-activation, initiation of FRET occurred in the regions of the NERs or poles (arrows) and proceeded laterally into the regions occupied only by the ER-derived vesicles. At 15 minutes the progression of the FRET signal can be seen in Supporting Information Movie S2. By 45–60 minutes a maximum FRET signal was attained around the entire nucleus as a complete envelope was formed by successive vesicle fusions, The time for enclosure is consistent with previous determinations [3]. Fig. 1B plots donor lifetime in the polar and equatorial quadrants as the reaction proceeds. By 5 minutes the donor lifetime decreased from 3.2 to 2.5±0.15 ns at the poles, remaining unchanged in the equatorial regions. Upon completion of fusion the entire nuclear envelope had a lifetime of 2.5±0.15 ns and a FRET efficiency of 0.7. Thus MV2 vesicle fusion apparently does not proceed unless adjacent to previously fused vesicles.

**Figure 2:**
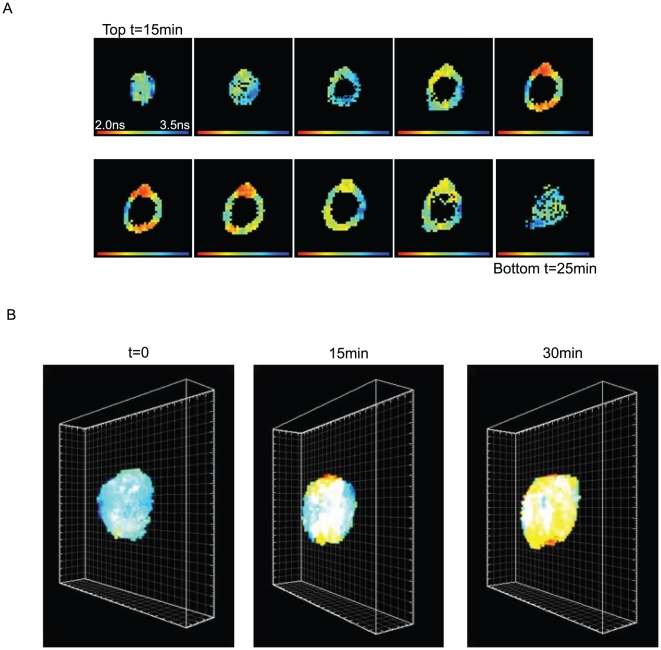
3D view of nuclei visualised by FLIM. (A) Stack measurements of a nucleus: the first image corresponds to the fluorescence lifetime of the BODIPY measured at the top of a nucleus 15 minutes after photo-activation of caged GTP. Ten successive layers of the same nucleus were obtained. For each layer the focus of the objective was moved 0.25 µm along the Z-axis. Since the acquisition of one image lasts for 1 minute, the last image corresponding to the bottom of the nucleus was measured 25 minutes after the induction of NE formation. (B) 3D reconstructions from the Z-stacks of the same nucleus to form fluorescence lifetime 3D views. The indicated times correspond to the time elapsed after photo activation when the first image of each stack was measured.

To visualise the polarised progression of fusion in three dimensions, confocal Z-stacks were obtained and a 3-D representation was reconstructed. Fig. 2 shows initiation sites of fusion in the middle set of stacks containing the NERs and the polarised progression of fusion to almost entirely envelop the spherical nucleus by 30 minutes (Supplementary Information Movie S3).

**Figure 3 pone-0012208-g003:**
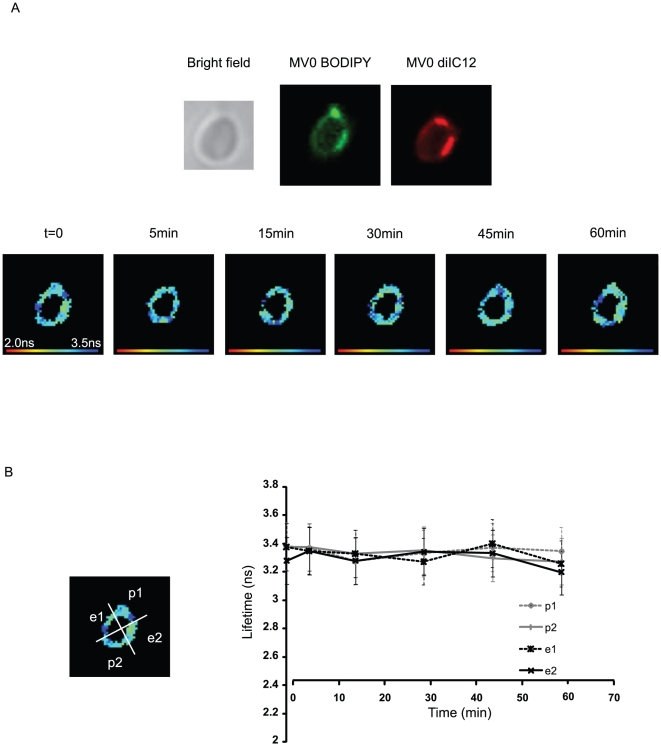
Inhibition of PLC prevents membrane fusion. The same experiment as in Fig. 2 was carried out in the presence of 30 µM U73122, a specific PLC inhibitor. The images (A) and lifetime graph (B) show complete inhibition of MV fusion. Data representative of 3 independent experiments

Substitution of non-hydrolysable GTPγ-S inhibited fusion (Fig. S2). To show that the GTP-initiated fusion was effected by the hydrolysis of PtdIns(4,5)P_2_, the PLC inhibitor U73122 was included. Fig. 3 shows that inhibition of PLC prevented the decrease of the donor lifetime, which remained at 3.3±0.16 ns.

**Figure 4:**
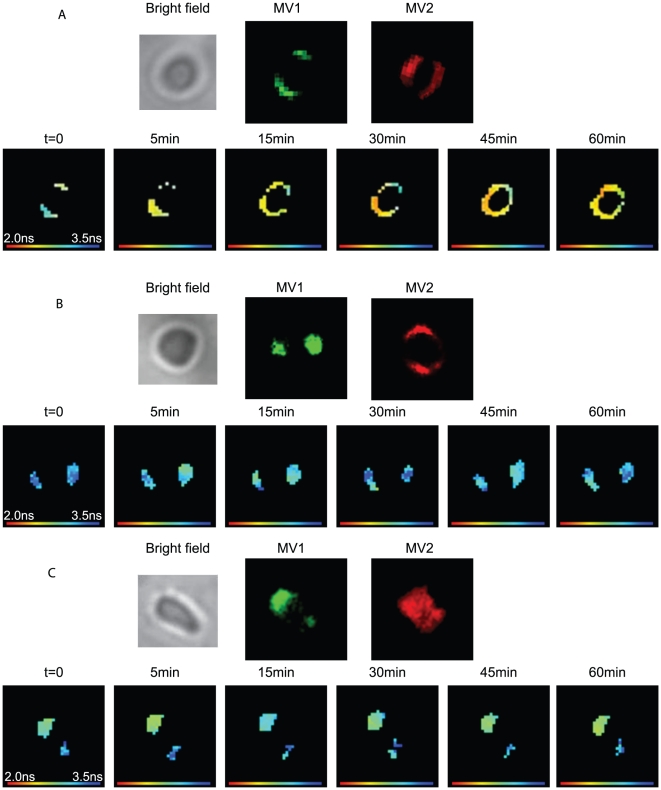
DAG is required for vectorial progression of fusion. (A) The same experiments as in Fig. 2 were performed using non-ER vesicles (MV1) labelled with BODIPY-C12 and ER vesicles (MV2) labelled with diIC12. Membrane fusion induces both a decrease of the lifetime and a spreading of BODIPY-C12 all around the nucleus. (B) Same experiment as in Fig. 4A carried out in the presence of 30 µM of U73122, indicating that fusion of the non-ER with the ER membranes requires PLC activity. (C) Same experiment as Fig. 4A using SKIP pre-treated MV1 vesicles. Dephosphorylation of PtdIns(4,5)P_2_ to PtdIns(4)P inhibits fusion.

The reactions presented thus far measure fusion between all nuclear bound vesicles (MV0) which include the minor population MV1 and the major population from ER (MV2). To show that initiation of membrane fusion at the poles results from PLCγ hydrolysis of PtdIns(4,5)P_2_ in the bound MV1 vesicles, we purified both MV1 and MV2, which were separately labelled with either the donor or acceptor fluorophore. Fig. 4A shows MV1 binding at the poles and MV2 over the rest of the chromatin surface. Fusion of MV1 with MV2 was initiated by GTP hydrolysis from the MV1 region (decrease of donor lifetime from 3.3 ns to 2.4 ns). By 15 minutes the fusion wave started to spread laterally to MV2. When MV1 was pre-treated with the PLC inhibitor U73122, membrane fusion was blocked (Fig. 4B). The inactive U73343 analogue did not prevent fusion (Fig. S4).

If MV1 was pre-treated with a recombinant phosphoinositide 5-phosphatase (SKIP) to deplete the substrate for PLC, membrane fusion was also prevented (Fig. 4C). SKIP dephosphorylates PtdIns(4,5)P_2_ to PtdIns(4)P which is not recognised by PLCγ and thus DAG cannot be produced. We show in Fig 4C a sperm nucleus which was not completely decondensed so its conical shape clearly defines the apical and basal poles. Pre-treatment of MV0 with SKIP also inhibited membrane fusion as expected (Fig. S3A). In a control parallel reaction, denatured SKIP failed to block membrane fusion and nuclear envelope formation (Fig. S3B).

Inhibition results are summarised in Table 1. These results strongly support our model of nuclear envelope formation involving GTP regulation of PLC hydrolysis of PtdIns(4,5)P_2_ [3], [4], [5] and add for the first time confirmation of the prediction of bipolarised spatial control of fusion initiation.

**Table 1:** Summary of membrane fusion effectors and inhibitors.

Treatment	Fusion
ATP	−
GTP	+
GTPγS	−
U73122 + GTP	−
U73343 + GTP	+
SKIP all MVs + GTP	−
SKIP non-ER MVs + GTP	−
Boiled SKIP all MVs + GTP	+

MVs independently labelled with either BODIPY-C12 (donor) or diIC12 (acceptor) were bound *in vitro* to sperm nuclei with ATP-GS (ATP). MV fusion was induced by photo activation of caged GTP and assessed by FLIM measurements as presented in Fig. 2, 3 and 4. The inhibitors U73122, U73343 (30 µM) or GTPγ[S] (2 mM) were applied just before mounting the sample and after 15 minutes of incubation GTP was activated. For inhibition of PtdIns(4,5)P_2_ phosphatase, MVs (either all MVs or only non-ER MV1) were incubated with SKIP purified proteins for 1 hour at room temperature prior to binding.

## Discussion

In this paper we provide evidence that fusion initiation leading to nuclear envelope formation is GTP triggered, and requires PtdIns(4,5)P_2_ and PLC activity in the MV1 fraction. Fusion is propagated bi-directionally from the sites of initiation and involves fusion of MV1 with MV2 as well as successive fusions with more MV2 to complete NE formation in the cell-free assay. The properties of the non-ER derived MV1 therefore are consistent with its role as a potentially fusogenic vesicle population regulated by GTP hydrolysis and resulting in heterotypic fusion with other vesicles by localised production of DAG.

These results lead to several questions: what is the origin of the polarisation, how is fusion propagated after initiation, and is the mechanism for fusion a general one? We discuss and offer speculation on these questions in turn below.

Since non-ER derived MV1 is a major source of the fusogenic DAG (and location of PLCγ in these cells), its restricted binding to the apex and base of the sperm nucleus lead to bi-directionality of fusion. The specificity of binding to the NER regions is likely to result from non-random packing of chromosomes in the sperm nucleus. MV1 binding requires NERs [18] which themselves have an unusual lipid composition and physical properties [19]. We have shown regions with similar properties present in the sperm nuclei of a wide range of animals suggesting they serve as nuclear membrane organising regions [20]. It remains to be demonstrated that each chromosome contains one or a few such structures which might also be used during mitotic NE reassembly.

Our data show that the mechanism of propagation of fusion commences from the polar MV1vesicles, which fuse with ER-derived vesicles. Subsequently ER vesicles fuse with one another towards the equatorial regions. The nuclear envelope precursor vesicles *in vitro* and *in vivo* are approximately 0.5 µm and the nucleus about 4 µm in diameter. Surface area calculations suggest that minimally 125 vesicles must fuse to envelop the nucleus with two bilayers [21]. If each NE were initiated by a single MV1 vesicle at the poles, the lipid contribution of MV1 to the completed nuclear envelope could be <1% of the total. Since nearly all PLCγ is associated with MV1 and is >100-fold enriched in MV1 as well as its substrate PtdIns(4,5)P_2_ [3], it is likely that DAG formed in this compartment rapidly diffuses into the ER-derived membranes with successive fusions, continually lowering the DAG concentration until it is below the 4% estimated from synthetic systems to be required for fusion [22]. Due to this dilution, eventually the fusogenicity of the forming envelope towards successive vesicles would likely decrease, and the process might become self-limiting. To determine if a decrease in the rate of successive fusions accompanies propagation requires a much a higher resolution method than used here.

The non-ER derived MV1 vesicle fraction enriched in PLCγ is found in vesicles in the cortex of oocytes [3] whereas the ER *in vivo* is usually a continuous membrane structure [23] that is of necessity vesiculated during preparation of cell extracts. *In vivo*, it is likely that tubules or continuous sheets of ER envelop the chromosomes at telophase. Thus the role of the non-ER fusogenic MV1 vesicles would be to seal gaps in the enveloping ER [5]. Local DAG concentrations could decline through dilution, chemical modification or both, returning the membrane to a non-fusigenic state.

A complete description of the spatial rearrangements of MV1 during the cell cycle has yet to be made. It is however clear that there are many more non-ER vesicles enriched in PLCγ than are necessary to facilitate NE formation [21]. It will therefore be of interest to determine whether such potentially fusogenic vesicles are mobilised to participate in other membrane fusion events during mitosis or interphase [24]. A novel aspect of this mechanism is that the specificity of localisation of DAG would depend on its delivery through the recruitment of potentially fusogenic vesicles to the sites of fusion rather than by its generation within a domain of one or both partner membranes to be fused.

We have shown that membrane fusion can involve a vectorial progression with a specific origin and direction. This vectorial progression is dependent on the formation of localised DAG derived from polyphosphoinositide modification in one of the partner membranes. The interplay of localised lipids and protein recruitment will be of importance to explore in a variety of natural membrane fusions.

## Supporting Information

Methods S1 (0.10 MB DOC)

Figure S1 Confocal images of sperm nuclei, nuclear envelope remnants and bound MVs. (A) Input sperm nuclei (extracted with 0.1% Triton X-100). DNA stained with propidium iodide [23] and detergent resistant membranes of the nuclear envelope remnants stained with diOC6 located at the acrosomal and centriolar fossa regions (apex and base) of the undecondensed nucleus. (B) Decondensed sperm nucleus with bound membrane vesicles from fertilised egg extract (S10). DNA stained with Hoechst 33334 (blue) and membranes stained with diOC6 (green). Nuclei incubated in S10 with ATP have decondensed chromatin and bound vesicles. (C) Nuclei with bound vesicles separated from unbound vesicles remaining in the S10 following purification through 0.5 M sucrose. The majority of bound vesicles are from the ER-derived population (MV2), which binds over the entire surface. The binding of non-ER derived membrane vesicle (MV1) occurs only in the regions of the NERs.
(2.26 MB EPS)

Figure S2 GTPγS does not induce fusion. The same experiment presented in Figure 1 was carried out using GTPγS instead of caged GTP. Under these conditions the lifetime of the donor remains constant indicating that there is no fusion of the labelled vesicles.
(0.57 MB EPS)

Figure S3 Pre-treatment of MVs with SKIP inhibits the fusion process. (A) Total S10 membranes (MV0) were pre-treated with SKIP as described in Experimental Procedures. Pre-treated membranes were independently labelled with either BODIPYC12 (donor) or diIC12 (acceptor) and both bound in vitro to sperm nuclei with ATP-GS (ATP). MV fusion was induced by photo activation of caged GTP and assessed by FLIM measurements as presented in Figures 2 to [Fig pone-0012208-g003] 4. (B) As a control the same experiment as in (A) was performed with denatured SKIP (5 minutes at 100oC). Denaturation of SKIP abolished the inhibition of fusion.
(0.88 MB EPS)

Figure S4 U73343, an inactive analogue of U73122, does not inhibit fusion. To confirm the specificity of the U73122 inhibitor, the same experiment as presented in Figure 4 was preformed using the U73343 analogue. No inhibition of fusion was observed.
(0.88 MB EPS)

Movie S1 3D Movie of nuclei visualised by FLIM at 0 minutes prior to activation of caged GTP.
(7.65 MB MOV)

Movie S2 3D Movie of nuclei visualised by FLIM at 15 minutes after photo-activation of caged GTP.
(7.76 MB MOV)

Movie S3 3D Movie of nuclei visualised by FLIM at 30 minutes after photo-activation of caged GTP.
(7.71 MB MOV)
