# Supplementary material for: Spatial Regulation of Membrane Fusion Controlled by Modification of Phosphoinositides
Source: PLoS One. 2010 Aug 17;5(8):e12208. doi: 10.1371/journal.pone.0012208 (PMC2923163; doi:10.1371/journal.pone.0012208)
Supplement: Methods S1 — (0.10 MB DOC) [file pone.0012208.s001.doc]

**Methods S1**

**Material and Methods:**

**Fluorescence lifetime imaging microscopy (FLIM)**

Photobleaching was evaluated by comparing the photon count over time, maintaining the photobleached fraction < 2% in all the acquisitions. Data analyses were performed using in-house software developed by Paul Barber (Kings College). FRET measurements were based on the reduction of the fluorescence lifetime of the donor upon specific quenching with the acceptor. The fluorescence lifetime of the donor (τD) was obtained by fitting the intensity decay with a mono or a bi-exponential fit. In all our experiments, due to the low number of photons collected, a better fit (based on χ² determination) was obtained with a mono-exponential fit.

It should be noticed that the emission of the diIC12 is within the bandpass emission filter used for FLIM that might affect the quality of the lifetime determination. For this reason we did not discuss the lifetime variations in terms of quantitative distance between the probes. However, we have checked that there was no direct excitation of the acceptor so the variations of the lifetime over time remain indicative of FRET between the donor and the acceptor. The more the lifetime decreases, the more efficient is FRET (due to a decrease of the donor lifetime and to the collection of a few photons emitted by the donor).

**Fluorescence characterisation of BODIPY-C12 and diIC12.**

The fluorescence excitation and emission spectra of probes inserted in lipid vesicles (1% mol/mol) are presented in the left panel. The overlap of the emission spectrum of BODIPY-C12 with the excitation spectrum of DIC12 allows FRET to occur as soon as the probes are in close proximity.

The right panel is the fluorescence intensity measured after exciting samples at 488nm. Vesicles labelled with 1% BODIPY-C12 mixed with vesicles labelled with 1% diIC12 (plain line) show a high fluorescence of the donor (BODIPY-C12) and almost no fluorescence of the acceptor (diIC12). This indicates that there is no FRET when probes are inserted in different vesicles. In other experiments 1% of both BODIPY-C12 and diIC12 were inserted in the same lipid vesicles (dashed line). We observed a decrease of the fluorescence of the donor concomitant with an increase of the signal of the acceptor which are typical of FRET. In another set of experiments, the lifetime of the excited state of BODIPY-C12 was measured for similar preparations. This lifetime was 3.6±0.09ns in the presence of FRET and decreased to 1.7±0.07ns in the absence of FRET.

Vesicles labelled with BODIPY-C12 mixed with vesicles labelled with diIC12 were kept at room temperature for 2 hours and did not show any change in fluorescence intensity nor lifetime of the donor indicating that there is neither spontaneous fusion of the vesicles nor exchange of the fluorescent probes between vesicles.
